# Comparative adipose transcriptome analysis digs out genes related to fat deposition in two pig breeds

**DOI:** 10.1038/s41598-019-49548-5

**Published:** 2019-09-09

**Authors:** Kai Xing, Kejun Wang, Hong Ao, Shaokang Chen, Zhen Tan, Yuan Wang, Zhao Xitong, Ting Yang, Fengxia Zhang, Yibing liu, Hemin Ni, Xihui Sheng, Xiaolong Qi, Xiangguo Wang, Yong Guo, Chuduan Wang

**Affiliations:** 1 Animal Science and Technology College, Beijing University of Agriculture, Beijing, 102206 China; 2 Key Laboratory of Animal Genetics, Breeding and Reproduction, Ministry of Agriculture, National Engineering Laboratory for Animal Breeding, College of Animal Science and Technology, China Agricultural University, Beijing, 100193 China; 3 College of animal science and veterinary medicine, Henan Agricultural University, Zhengzhou, Henan 450002 China; 4 State Key Laboratory for Animal Nutrition, Key Laboratory for Domestic Animal Genetic Resources and Breeding of the Ministry of Agriculture of China, Institute of Animal Science, Chinese Academy of Agricultural Sciences, Beijing, 100193 China; 5 Beijing General Station of Animal Husbandry, Beijing, 100125 China

**Keywords:** Gene expression profiling, Gene expression

## Abstract

Fatness traits are important in pigs because of their implications for fattening efficiency, meat quality, reproductive performance and immunity. Songliao black pigs and Landrace pigs show important differences in production and meat quality traits, including fatness and muscle growth. Therefore, we used a high-throughput massively parallel RNA-seq approach to identify genes differentially expressed in backfat tissue between these two breeds (six pigs in each). An average of 37.87 million reads were obtained from the 12 samples. After statistical analysis of gene expression data by edgeR, a total of 877 differentially expressed genes were detected between the two pig breeds, 205 with higher expression and 672 with lower expression in Songliao pigs. Candidate genes (LCN2, CES3, DGKB, OLR1, LEP, PGM1, PCK1, ACACB, FADS1, FADS2, MOGAT2, SREBF1, PPARGC1B) with known effects on fatness traits were included among the DEGs. A total of 1071 lncRNAs were identified, and 85 of these lncRNAs were differentially expressed, including 53 up-regulated and 32 down-regulated lncRNAs, respectively. The differentially expressed genes and lncRNAs involved in glucagon signaling pathway, glycolysis/gluconeogenesis, insulin signaling pathway, MAPK signaling pathway and so on. Integrated analysis potential trans-regulating or cis-regulating relation between DEGs and DE lncRNAs, suggested lncRNA MSTRG.2479.1 might regulate the expressed level of VLDLR affecting porcine fat metabolism. These results provide a number of candidate genes and lncRNAs potentially involved in porcine fat deposition and provide a basis for future research on the molecular mechanisms underlying in fat deposition.

## Introduction

The pig (*Sus scrofa*) is an important domesticated species that provides more than 40% of the total meat consumed by humans globally^1^. As they exhibit optimal productivity and efficiency, modern European pig breeds are used in most production^2^. The Landrace pig is a typical lean-type modern breed and is now used for pork production worldwide. It exhibits high growth rates and lean carcass percentages^3^. In contrast, Songliao black pigs are characterised by higher fat deposition, fat desaturation and lower feed utilisation efficiency compared with European pig breeds (including Landrace pigs). Thus, comparison of these two pig breeds can be useful for studying porcine fat deposition and growth performance. The pig in general also an ideal biomedical model, such as of obesity and cardiovascular diseases, owing to the similarities in physiology, anatomy and genomic structure with humans^4^.

Fatness traits such as backfat deposition have been intensively studied in pigs owing to their strong relationship with growth performance, as the lower the level of deposited fat, the better the growth performance^5^. Variation in fat deposition, especially the subcutaneous, intermuscular (carcass fat) and intramuscular fat (IMF) content (marbling), also makes a major contribution to various aspects of meat quality and is central to the nutritional value of meat^6^. Adipose is the major site of energy storage in the form of lipids. Excess fat is stored in adipocytes of the adipose tissue. Adipose is also the site of de novo fatty acid synthesis in pigs^7^. Apart from storing lipids, adipose tissue functions as an endocrine organ that produces and secretes adipocytokines such as TNFα; peptide hormones such as leptin, adiponectin, oestrogen and resistin; and lipid hormones such as palmitoleate, which are involved in energy metabolism and inflammation^8^.

To characterise and quantify the global changes in the transcriptome, RNA-seq is one of the preferred approaches. RNA-seq can provide precise data on transcript levels and their isoforms or alternative splice sites, and enables the detection of transcripts expressed at low levels^9^. Recent studies have employed RNA-seq to analyse the transcriptome of porcine adipose tissue, focusing on differences among breeds^10,11^ and in backfat thickness^12,13^, fatty acid composition^14^, developmental period^15^ and storage position^16^. However, the function and molecular regulatory mechanism of adipose tissue in pigs are not clearly understood.

Long non-coding RNAs (lncRNAs), most of which longer than 200 base pairs, have been acted as a novel class of regulatory molecules involved in various biological processes^17^. In many studies, lncRNAs were shown to regulate gene expression program in several levels, including mRNA expression and degradation^18^, the effective concentration of miRNAs^19^ and DNA methylation^20^. Genome-wide analyses of lncRNA, always followed mRNA expression profile, have been performed to reveal phenotypic variation of fat deposition in pigs^21–24^. Several lncRNAs, such as SRA^25^, lnc-ADINR^26^, lncRNA-ADNCR^27^, lnc-BATE1^28^ and so on, were identified affecting adipocyte differentiation.

In the present study, RNA-seq technology was performed to analyse the transcriptome of adipose tissue from two pig breeds between which there are some significant phenotypic variations. Then, differential expressed genes and lncRNAs were detected and subjected to biofunctional analysis to reveal the differences between the breeds in genes and pathways that could be related to fatness traits. This study focuses on the identification of candidate genes and lncRNA that influence fatness traits and provides crucial regulatory information on the molecular mechanisms of adipose deposition in Landrace and Songliao black pigs.

## Results

### Analysis of RNA-seq data

A total of 12 cDNA libraries for backfat tissues from the two pig breeds with six biological replicates each were sequenced using HiSeq 2000. After filtering the adaptor and low-quality reads, 37.87 ± 1.74 million 90-base-pair (bp) paired-end reads were yielded. The mapped reads aligned to the porcine genome (Sscrofa 11.1) accounted for a mean of 92.81% of the total clean reads, including averages of 67.07%, 2.79%, 11.76%, and 14.21% of clean reads within coding sequences (CDSs), 5′-untranslated regions (UTRs), 3′-UTRs and introns regions, respectively. The distribution of the annotated reads was similar across individual samples in these two breeds. The deep sequencing data of total RNA have been submitted to NCBI Sequence Read Archive (SRA) under Bioproject: PRJNA234335 and Bioproject: PRJNA287471.

### Gene expression analysis

**Figure 1:**
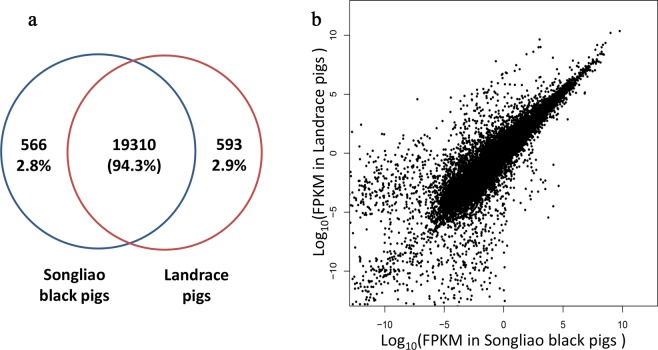
Gene expression levels in backfat tissue for Songliao black pigs and Landrace pigs. (**a**) Venn diagrams show the total number of expressed genes in the two pig breeds. The number of common genes is shown in the overlapping segments. (**b**) The x- and y-axes plot the gene expression counts in Songliao black and Landrace pigs after FPKM quantification, respectively.

**Figure 2:**
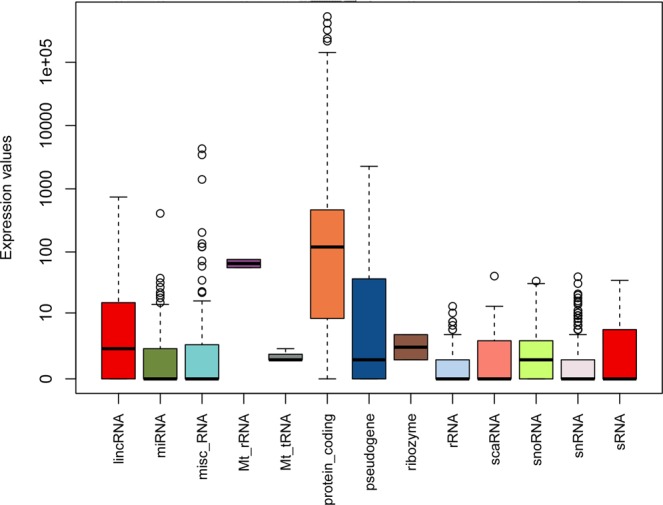
Count distribution per biotype in all samples.

The total number of expressed genes in adipose tissue of different samples ranged from 17,212 to 18,547 and showed no significant difference between two breeds (Table S1). There was very high reproducibility for gene expressed level between the two breeds, indicating that majority of the adipose transcriptome appears to be conserved between them (Fig. 1). In this study, non-coding RNAs were also identified. Count numbers of each type RNAs were shown in Fig. 2.

A number of genes have highly expressed in adipose tissue of the two breeds, such as Cytochrome C oxidase subunit 1 (COX1), Cytochrome C oxidase subunit 3 (COX3), Collagen type I alpha 1 chain (COL1A1), ATP synthase F0 subunit 6 (ATP6), Cytochrome C oxidase subunit 2 (COX2), Fatty acid synthase (FASN), NADH-ubiquinone oxidoreductase chain 4 (ND4), collagen type III alpha 1 (COL3A1), and Cytochrome b (CYTB). These are the 10 most highly expressed genes in the samples and thus could play important roles in adipose tissue.

**Figure 3:**
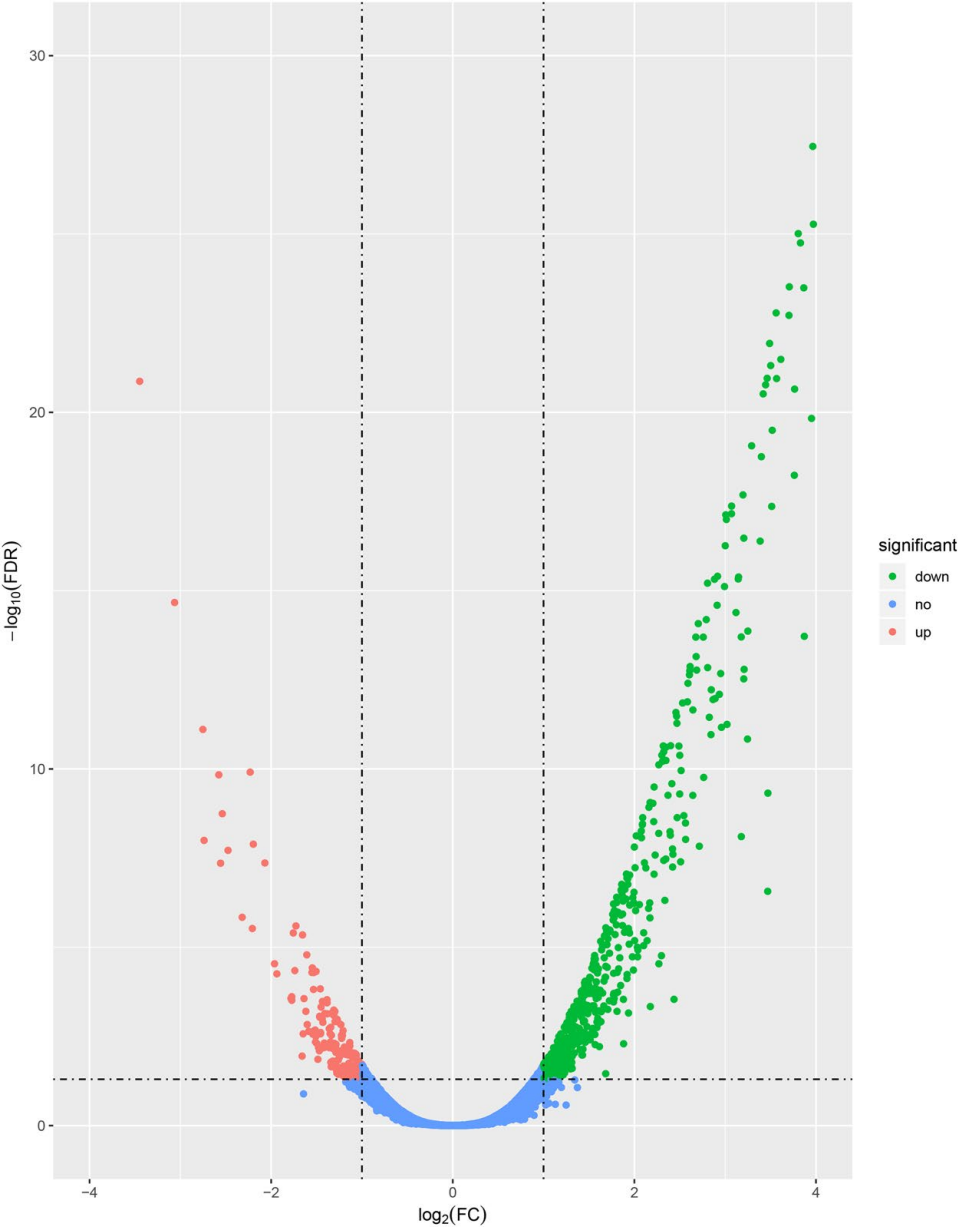
Volcano plot of genes differentially expressed between Songliao black and Landrace pigs. The y-axis corresponds to the mean expression value of log10(q-value), and the x-axis displays the log_2_ (FC) value. The red dots represent the significantly overexpressed genes in Songliao groups (FDR < 0.05, FC ≥ 2); The green dots represent the significantly underexpressed genes in Songliao groups (FDR < 0.05, FC ≤ 0.5); the blue dots represent the genes whose expression levels did not reach statistical significance.

To select genes potentially strongly associated with fatness traits, the genes with differential expression in adipose between Songliao black pigs and Landrace pigs were identified. RNA-seq data analysis detected differential expression of 877 genes using edgeR (fold change (FC) ≥ 2 or FC ≤ 0.5, and FDR < 0.05), including 205 (23.38%) genes that were down regulated and 672 (76.62%) that were downregulated in Songliao pigs. Of these, 65 genes are novel and 812 genes are known. The details of all DEGs found in the compared breeds (Landrace and Songliao pigs) were shown in Supplementary Table S2, and all genes expressed information in the two comparison groups was depicted in volcano plot (Fig. 3). In consideration of research aim, we investigated 98 DEGs related to lipid metabolism and adipogenesis (Table S3). Several significantly upregulated genes in Landrace group such as lipocalin 2 (LCN2), carboxylesterase 3 (CES3), diacylglycerol kinase beta (DGKB), oxidized low density lipoprotein receptor 1 (OLR1), hydroxysteroid 17-beta dehydrogenase 14 (HSD17B14), leptin (LEP) and so on participate in lipids and energy metabolism. Those RNA-seq results reaffirmed the significantly upregulated genes in Songliao group including phosphoglucomutase 1 (PGM1), phosphoenolpyruvate carboxykinase 1 (PCK1), acetyl-CoA carboxylase beta (ACACB), fatty acid desaturase 1 (FADS1), fatty acid desaturase 2 (FADS2), monoacylglycerol O-acyltransferase 2 (MOGAT2) and so on play crucial roles in *de novo* biosynthesis of fatty acid. In addition, sterol regulatory element binding transcription factor 1 (SREBF1) and PPARG coactivator 1 beta (PPARGC1B), two important transcription factors controlling cellular lipogenesis, were observed to be differentially expressed. Moreover, list of the most significant 15 differentially expressed genes between Songliao black and landrace pigs were shown in Table 1.

**Table 1:** List of the most significant 15 differentially expressed genes in Songliao black and landrace pigs.

Ensembl ID	Log_2_(FC)	*P*-value	*Q-*value	Gene symbol
ENSSSCG00000011646	8.02	2.59E-70	1.68E-66	KY
ENSSSCG00000011508	6.93	5.90E-58	1.91E-54	LMOD3
ENSSSCG00000010303	7.24	1.49E-57	3.87E-54	SYNPO2L
ENSSSCG00000014443	7.02	3.75E-57	8.11E-54	CAMK2A
ENSSSCG00000029471	6.50	7.68E-57	1.42E-53	STAC3
ENSSSCG00000007231	7.35	9.91E-55	1.43E-51	MYLK2
ENSSSCG00000014128	7.55	2.38E-54	3.08E-51	CKMT2
ENSSSCG00000011441	6.68	4.26E-53	5.02E-50	TNNC1
ENSSSCG00000039710	8.02	1.16E-52	1.25E-49	MYL2
ENSSSCG00000007757	6.16	2.07E-52	2.06E-49	TRIM72
ENSSSCG00000006754	8.42	2.04E-50	1.89E-47	AMPD1
ENSSSCG00000006174	6.06	5.06E-50	4.16E-47	JPH1
ENSSSCG00000014324	9.54	5.13E-50	4.16E-47	MYOT
ENSSSCG00000025353	7.54	1.05E-49	7.94E-47	TNNT1
ENSSSCG00000001806	5.66	6.24E-49	4.26E-46	FSD2

### Functional enrichment analysis of differentially expressed genes

**Figure 4:**
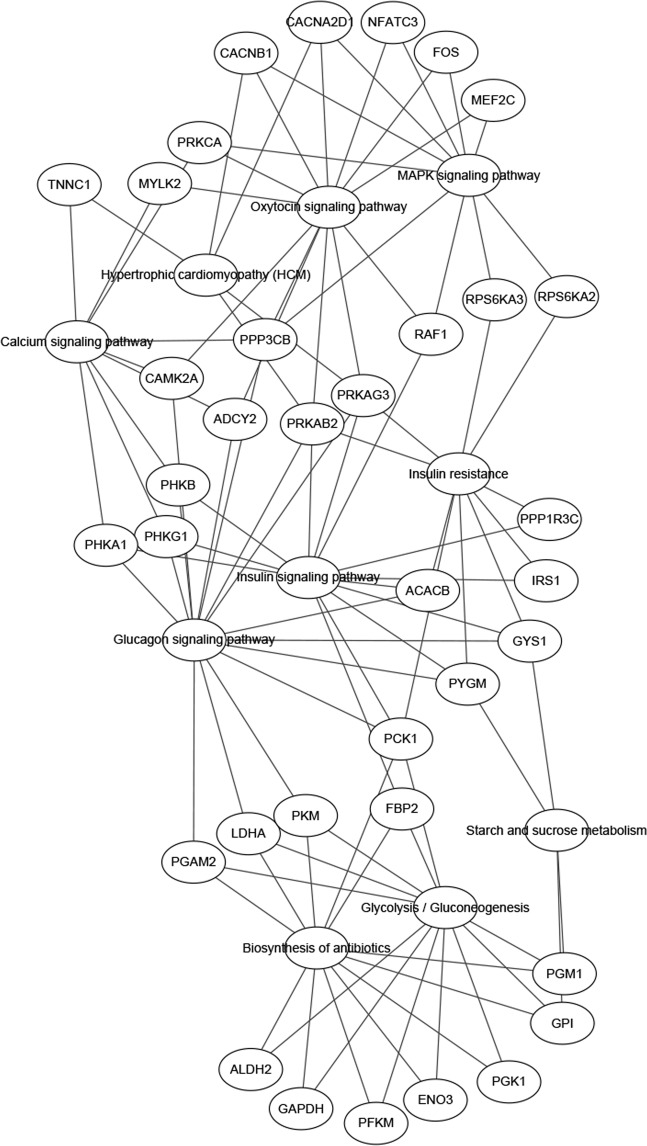
Putative metabolic network regulated by DEGs and significantly enriched pathways.

To obtain insight into the biological relationships of genes that were differentially expressed in adipose between Songliao black pigs and Landrace pigs, we performed gene ontology (GO) biological process and Kyoto Encyclopedia of Genes and Genomes (KEGG) pathway enrichment analyses. The GO analysis results showed that DEGs were significantly enriched in 27 GO terms (Table S4). These included gluconeogenesis (GO: 0006094), glycogen catabolic process (GO:0005980), glycolytic process (GO: 0006096) and ATP binding (GO: 0005524), which are involved in fat metabolism or energy metabolism. Significantly enriched KEGG pathways (P < 0.05) were also identified, which included glucagon signaling pathway, glycolysis/gluconeogenesis, insulin signaling pathway, MAPK signalling pathway, glucagon signalling pathway, oxytocin signaling pathway, insulin resistance, and starch and sucrose metabolism (Table 2). The network of DEGs and KEGG pathways were shown in Fig. 4.

**Table 2:** Significantly enriched pathways analysis for differentially expressed genes.

Pathway	count	P-Value^a^	Genes
Glucagon signaling pathway	15	2.9E-3	PYGM, LDHA, PHKA1, PGAM2, PRKAG3, PPP3CB, PHKB, GYS1, CAMK2A, ACACB, PRKAB2, ADCY2, PCK1, PKM, PHKG1
Glycolysis/Gluconeogenesis	12	3.0E-3	LDHA, GAPDH, PGAM2, PGK1, FBP2, GPI, ENO3, PCK1, PKM, ALDH2, PGM1, PFKM
Insulin signaling pathway	17	3.0E-3	PYGM, PHKA1, RAPGEF1, RAF1, IRS1, GYS1, ACACB, ARAF, PPP1R3C, PHKG1, PRKAR2A, EIF4E, PRKAG3, PHKB, FBP2, PRKAB2, PCK1
MAPK signaling pathway	24	2.9E-3	MAP2K6, RPS6KA2, EGF, NFATC3, RAF1, MAP3K2, SRF, PPP3CB, MEF2C, FOS, PPM1B, FGF13, CACNA2D1, GADD45B, FGFR4, FLNC, MAP3K20, HSPA1B, PS6KA3, MAPT, CACNB1, ECSIT, PRKCA, MAPKAPK2
Biosynthesis of antibiotics	20	1.2E-2	ARG2, PGAM2, AMPD1, DAO, IDH2, PHGDH, AK1, PKM, LDHA, GAPDH, AK5, PGK1, FBP2, GPI, ENO3, ALDH2, PCK1, DLST, PGM1, PFKM
Hypertrophic cardiomyopathy (HCM)	11	1.9E-2	PRKAG3, ITGA8, TNNC1, DES, TPM1, SGCD, PRKAB2, CACNB1, CACNA2D1, TPM2, SGCG
Oxytocin signaling pathway	16	2.0E-2	MYLK2, NFATC3, RAF1, PPP3CB, CAMK2A, MEF2C, FOS, ADCY2, CACNA2D1, RCAN1, ROCK2, PRKAG3, CAMK1G, PRKAB2, CACNB1, PRKCA
Calcium signaling pathway	17	2.3E-2	PLCE1, PHKA1, MYLK2, SLC25A4, PPP3CB, CAMK2A, ATP2A1, ADCY2, PHKG1, TNNC2, P2RX5, PHKB, PLCD4, TNNC1, PTGER3, ORAI3, PRKCA
Starch and sucrose metabolism	7	2.7E-2	PYGM, PGM2L1, TREH, GYS1, AGL, GPI, PGM1
Insulin resistance	12	4.2E-2	PYGM,RPS6KA2, PRKCQ, PPARGC1B, PRKAG3, IRS1, GYS1, ACACB, RPS6KA3, PPP1R3C, PRKAB2,PCK1

^a^The P-value after adjustment by the Benjamini.

### Identification of differentially expressed lncRNAs

**Figure 5:**
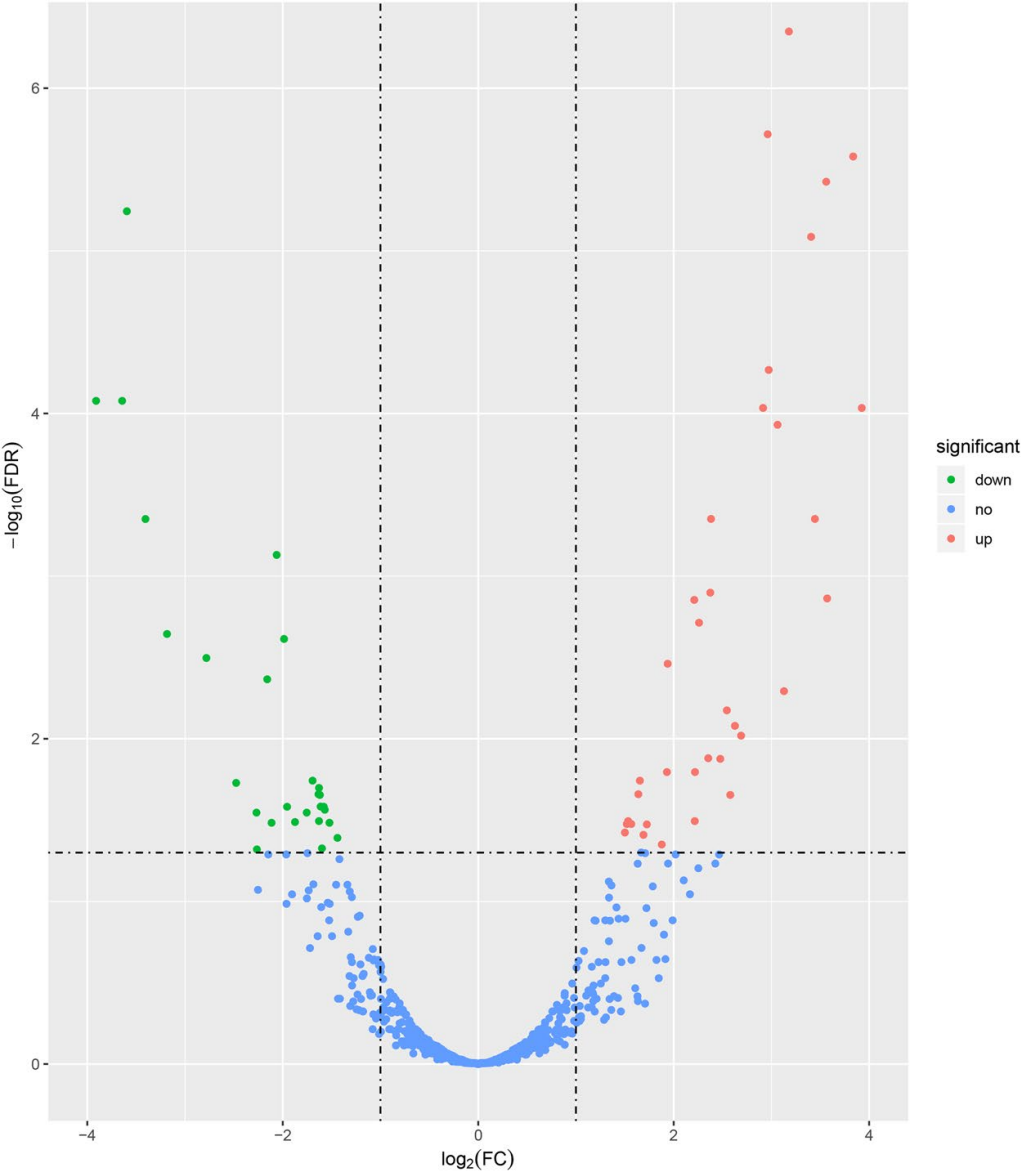
Volcano plot of long non-coding genes differentially expressed between Songliao black and Landrace pigs. The y-axis corresponds to the mean expression value of log_10_(q-value), and the x-axis displays the log_2_ (FC) value. The red dots represent the significantly overexpressed LncRNAs in Songliao groups (FDR < 0.05, FC ≥ 2); The green dots represent the significantly underexpressed LncRNAs in Songliao groups (FDR < 0.05, FC ≤ 0.5); the blue dots represent the genes whose expression levels did not reach statistical significance.

All clean data were mapped to the Sscrofa 11.1 genome assembly and an average of mapping rate more than 76.62%. A total of 61,465 transcripts were identified based on merging 12 files. Of these, 18,012 transcripts with class code “i”, “u”, “x” and “s” were retained. Then, transcript with length more than 200 bp, possed more than one exon, CNCI and CPC score less than zero were retained. After a serious of filtered steps, 1071 lnRNAs were identified (Table S5). By edgeR, 84 of those lncRNAs were significant differentially expressed between two groups (Table S6), including 52 up-regulated and 32 down-regulated genes, respectively (Fig. 5).

To detect cis-regulate the expression of genes located in their vicinity, 83 DE lncRNAs were found those mapped near (100 kb or less) to the location of neighboring target DEGs (Table S7). In added, all of 85 DE lncRNAs were identified having opposite transcriptional direction compared to differentially expressed genes (Table S8).

### DEGs in QTL database related to fatness traits and previous similar reports

QTLs related to fatness traits in an animal QTL database were selected for comparing with our results. A total of 54 DEGs overlapped with the regions of the chosen QTLs, identifying them as candidate genes that potentially affect fat deposition (Table S9). Twenty-two DEGs in our study were matched DEGs list in previous studies (Table S10), which may represent additional information candidate genes.

### Validation of DEGs using qPCR

**Figure 6:**
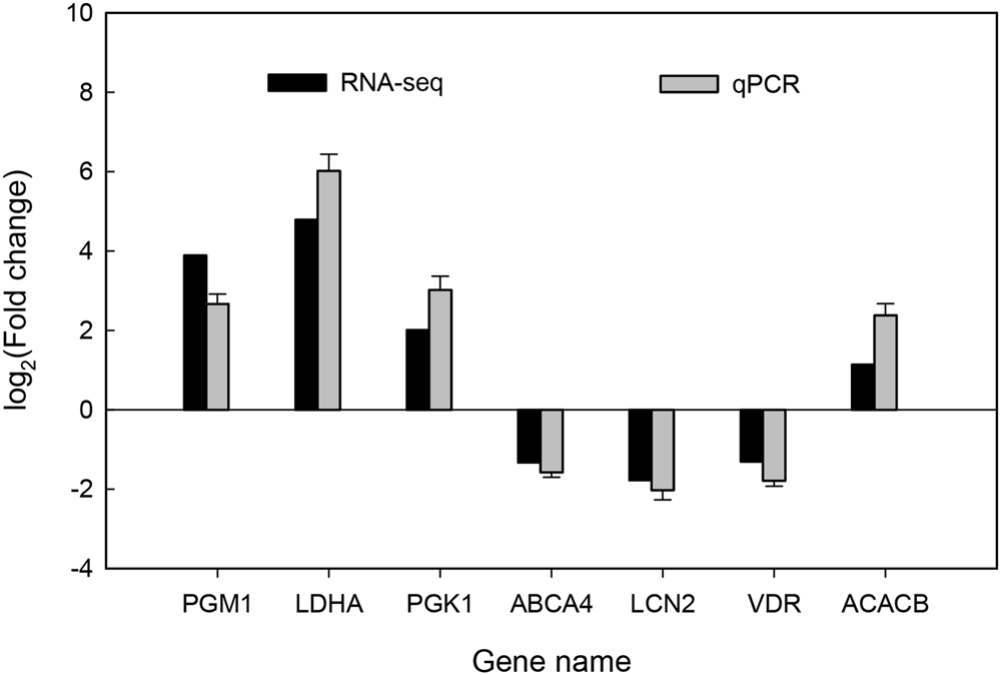
Validation of RNA-seq results by qPCR.

Seven DEGs, including four up-regulated genes (PGM1, LDHA, PGK1 and ACACB) and three down-regulated genes (ABCA4, LCN2 and VDR) in Songliao groups, were selected for qPCR to verify the information of DEGs obtained by RNA-seq. For all 7 DEGs, the direction of gene expression changes was similar between those measured by qPCR and RNA-seq analysis (Fig. 6).

## Discussion

The adipose tissue is a complex and metabolically active organ, and the adipocytes of which it is composed are a dynamic and highly regulated population of cells. Fat deposition in adipose is a complex metabolic process involving many genes, including coding and no coding gens. Paired expression profiling was used to investigate lncRNAs and mRNAs function in fat deposition. However, the regulatory mechanism of mRNA and lncRNAs in fat deposition remains poorly understood.

The results of our study support the status of adipocytes as highly complex cells that play important roles in various metabolic processes. Carbohydrate metabolism is an important part of energy metabolism and lipid metabolism. It is upstream of *de novo* fatty acid synthesis and can supply the substrates for FA. PGM1, a key enzyme in carbohydrate metabolism, catalyses the reversible conversion reaction between glucose-1-phosphate and glucose-6-phosphate. It also participates in the anabolic pathway that generates NADPH for the reductive biosynthesis of compounds such as fatty acids in adipocytes^29^. Consistent with our results, PGM1 has also been reported to be upregulated in pork with higher intramuscular fat^30^. Gluconeogenesis is the cycle through which glucose is used to produce fat or protein. In Landrace pigs, we observed higher mRNA expression of several glycolytic genes that are important in gluconeogenesis, including glucose-6-phosphate isomerase (GPI), phosphoglycerate kinase (PGK), lactate dehydrogenase A (LDHA), phosphoglucomutase 1 (PGM1) and phosphoglycerate mutase 2 (PGAM2). PGK, a glycolytic enzyme that catalyses the conversion of 3-phosphoglycerate into 2-phosphoglycerate, was more highly expressed in fat tissue of Songliao pigs in our study. This is an interesting finding as polymorphism of the PGK gene has been reported to have significant effects on weight at birth and weight at 3 weeks in pigs^31^. This suggests that the PGK gene could be associated with energy metabolism and growth in pigs. In addition, PGAM1, which was upregulated in adipose tissue of Songliao black pigs, catalyses the reversible reaction of 3-phosphoglycerate to 2-phosphoglycerate in the glycolytic pathway and plays an important role in coordinating energy production. LDHA participates in catalysing the conversion of L-lactate and NAD to pyruvate and NADH in the final step of anaerobic glycolysis^32^. It has also been reported that single-nucleotide polymorphism in the porcine LDHA gene was associated with the average daily gain in Italian Large White pigs^33^. These results indicate that carbohydrate utilisation and energy metabolism are probably very important in fat deposition. Pyruvate kinase M (PKM) is involved in glycolysis and catalyses the transfer of a phosphoryl group from phosphoenolpyruvate to ADP, generating ATP and pyruvate^34^. Polymorphisms of the PKM2 gene were previously reported to be associated with backfat thickness in Berkshire pigs^35^. Another example of a gene that involved in glycogenolysis in this study is Glycogen phosphorylase (PYGM)^36^.

Glucagon and insulin are conventionally regarded as counterregulatory hormones and play critical roles by maintaining glucose homeostasis in both animals and humans^37^. To increase blood glucose, glucagon promotes hepatic glucose output by increasing glycogenolysis and gluconeogenesis and by decreasing glycogenesis and glycolysis in a concerted fashion via multiple mechanisms^38^. From the KEGG pathway analysis in the current study, the glucagon signalling pathway, insulin resistance and insulin signalling pathway were found to be significantly associated with the DEGs between the two pig breeds. Several key genes of these pathways were found to be significantly differentially expressed in our study. The insulin receptor substrate 1 (IRS1) gene encodes a protein that is phosphorylated by insulin receptor tyrosine kinase. This protein is a key regulator in insulin signal transduction, in a manner dependent on its serine-to-threonine phosphorylation ratio, affecting insulin sensitivity^39^. Acetyl-CoA carboxylase-beta (ACACB) acts as the rate-limiting step in fatty acid uptake and oxidation by mitochondria through the control of fatty acid oxidation^40^. Conflicting with our results, the expression level of ACACB in obese pigs was found to be higher than that in lean ones^41,42^. Protein phosphatase 1 regulatory subunit 3C (PPP1R3C) and PPP1R3G are more ubiquitously expressed and are found in insulin-sensitive tissue, including skeletal muscle, liver and fat. It affects glycogen biosynthesis by activating glycogen synthase and limiting glycogen breakdown by reducing glycogen phosphorylase activity^43^.

Two genes in top significant 15 DEGs were also related to energy, glucose or lipid metabolism. Adenine monophosphate deaminase 1 (AMPD1) is an isoform of AMPD, which catalyze the deamination of AMP to monophosphate (IMP)^44^. AMPD1 plays a central role in the regulation of lipid and glucose metabolism through impacting of AMP-activated protein kinase (AMPK) activation^45^. Furthermore, it was reported that AMPD1 could leads to reduce insulin resistance, improved glucose tolerance and enhanced insulin clearance in mice fed a high fat diet^46^. Creatine kinase, mitochondrial 2 (CKMT2), higher expressed in Songliao black groups, is responsible for the transfer of high energy phosphate from mitochondria to cytoplasm^47^. As its higher enriched level in brown adipocyte (BAT), CKMT2 was described as markers of BAT^48^. Consistent of our results, the mRNA level of CKMT2 in fatten pigs was higher than lean pigs^49^. AMPD1 and CKMT2 could be novel promising candidate genes affecting fat deposition.

In our study, 1071 lncRNAs were detected in adipose tissue of Songliao and Landrace. The value doubles the number of detected lncRNAs in Duroc and Luchuan pigs^21^, but half of that in Jinhua and Landrace pigs^23^. Eighty-four differentially expressed lncRNAs were identified from six samples, including up-regulated lncRNAs and down-regulated lncRNAs. As potential trans-regulating or cis-regulating relation, several of these differentially expressed lncRNAs were predicated to participate in fat metabolism or deposition. lncRNA-MSTRG.2479.1 might regulate the expressed level of very low-density lipoprotein receptor (VLDLR), and VLDLR take part in triglyceride metabolism^50^. As above mentioned, ACACB is an important enzyme in fatty acid oxidation^40^, and it could be regulated by multiple DE lncRNAs. Thus, the obtained results indicated the potential regulatory functionalities of differentially lncRNAs in the fat deposition and fatty acid metabolism of pigs.

## Conclusions

In this study, a comparison of transcriptome was performed in Landrace and Songliao black pigs. A total of 877 differentially expressed genes were detected between the two groups. In line with their different phenotypes, these identified genes were found to participate in pathways related to fat metabolism, regulation and transport such as carbohydrate metabolism, glycolysis/gluconeogenesis, glucagon signalling pathway, insulin resistance, insulin signalling pathway and so on. A number of known genes involved in fat deposition, including LCN2, CES3, DGKB, OLR1, LEP, PGM1, PCK1, ACACB, FADS1, FADS2, MOGAT2, SREBF1, PPARGC1B and so on, were revealed. Moreover, AMPD1 and CKMT2 were considered to novel promising candidate genes affecting fat deposition. A total of 1071 lncRNAs were identified, and 85 of these lncRNAs were differentially expressed, including 53 upregulated and 32 downregulated lncRNAs, respectively. Integrated analysis potential trans-regulating or cis-regulating relation between DEGs and DE lncRNAs, suggested lncRNA MSTRG.2479.1 might regulate the expressed level of VLDLR affecting porcine fat metabolism. In summary, this study presents a number of candidate genes and lncRNAs for porcine fat deposition and provides a basis for future research on the molecular mechanisms underlying phenotypic differences in fat deposition.

## Methods

### Ethics statement

All protocols and procedures involving animals were performed in accordance with the Regulations for the Administration of Affairs Concerning Experimental Animals (Ministry of Science and Technology, China) and were approved by the Animal Welfare Committee of China Agricultural University (permit number: DK996). During the experimental period, the animals were reared in the same environment, were fed the same diet ad libitum and were humanely sacrificed.

### Animals and sample collection

Female Songliao and Landrace pigs formed the source population, which were provided by Tianjin Ninghe primary pig breeding farm (Ninghe, China). Six female pigs each from Landrace and Songliao black breeds were used in this study. Animals were reared under the same conditions with natural, uncontrolled room temperature and light. Animal were slaughtered in a commercial abattoir (Beijing Huadu Sunshine Food Co., Ltd., Beijing, China). All efforts were made to minimize animal suffering during the study. The adipose tissue of backfat was collected aseptically and quickly. Samples were stored immediately in liquid nitrogen until used for RNA extraction.

### RNA isolation, library preparation and sequencing

The total RNA from adipose tissue was isolated using the total RNA extraction kit (Bioteke, Beijing, China), in accordance with the manufacturer’s recommendations. The RNA quantity and quality were assessed with an Agilent 2100 Bioanalyzer (Agilent, Santa Clara, CA) and electrophoresis on a 1% agarose gel. For each sample, a cDNA library was synthesised according to the Illumina paired-end library preparation protocol (Illumina, San Diego, CA). Paired-end (PE) reads were obtained on an Illumina HiSeq 2000 sequencing system after sequencing.

### Data analysis

Data analysis included quality control, filtering, mapping, assembly and annotation of RNA-seq reads. CASAVA software (Illumina) was used to obtain raw sequence data in fastq format. FASTQC^51^ (http://www.bioinformatics.babraham.ac.uk/projects/fastqc/) was used to remove adaptor sequences, reads with unknown sequences “N” and low-quality sequences (those with a percentage of low-quality bases with a threshold quality score <20). Clean reads were aligned to the porcine reference genome sequence (Sscrofa 11.1) using TopHat v2.0.1 software^52^. Reads aligned to the reference genome were assembled using Cufflinks software^53^. HT-seq was used to count the number of annotated clean reads for each gene^54^. The expression of genes in different libraries was normalised by the Trimmed Mean of M-values (TMM)^55^, and DEGs (FC ≥ 2 or FC ≤ 0.5, and FDR < 0.05) were identified using the edgeR package^56^.

### Functional analysis of DEGs

Before the functional analysis of DEGs, the porcine Ensembl gene IDs were converted to homologous human Ensembl gene IDs using bioDBnet^57^ (https://biodbnet-abcc.ncifcrf.gov/). Kyoto Encyclopedia of Genes and Genomes (KEGG) pathway and Gene Ontology (GO) enrichment analyses of DEGs were performed using the Database for Annotation, Visualization and Integrated Discovery (DAVID)^58^ bioinformatic resource (http://david.abcc.ncifcrf.gov/). GO terms consisted of molecular functions (MF), biological processes (BP) and cellular components (CC). Fisher’s exact tests with the Benjamini (<0.05) were used to obtain an adjusted P-value in this analysis. Cytoscape software was used to plot the DEG-Pathway network^59^.

### Identification of differentially expressed lncRNAs

Clean pair-end reads were aligned to the Sscrofa 11.1 genome assembly with the Hisat2 (v2.0.0-beta). Bam files were subjected to StringTie (v1.3.0) and then gffcompare (v0.10.1) to identify novel tranascripts compared to Ensemble gene sets (Sscrofa 11.1). The transcripts whose class code annotated by “i,” “u,” “x,” and “s” were retained and subjected to lncRNA identification. To avoid incomplete assemble and too may splicing events, the putative lncRNA with transcripts length >200 nt and exon number >2 were retained. Remaining transcripts were predicted protein-coding potential were removed using CNCI tool (score < 0)^60^ and CPC (Coding Potential Calculator)^61^. We used HTseq^54^ software to calculate the number of reads that mapped to each transcripts. edgeR package^56^ was used to identify the differential expressed transcripts with the criterion (FC ≥ 2 or FC ≤ 0.5, and FDR < 0.05). Bedtools software was used to identify the cis-regulating relationship between differentially expressed lncRNAs and mRNAs within upstream/downstream of 100 kb^62^. For trans-regulation targeting events, LncTar was applied to investigate the interaction between lncRNA and mRNA^63^.

### DEG comparison with the animal QTL database and previous transcriptome information

To identify candidate genes associated with porcine fatness traits, we integrated DEGs between the two breeds and QTL information for fat deposition from an animal QTL database^64^ (http://www.animalgenome.org/QTLdb) by comparing their chromosome positions. This database contains all publicly available QTL data on pigs and includes 25,610 QTLs, of which 1,741 are associated with fatness in pigs. The size of the published QTL region is also limited. Only those regions less than 1 Mb and related to fatness were considered as available QTLs. Because several previous studies analysed backfat transcriptomes from different breeding groups with diverging fatness traits or fatty/lean phenotypes, the DEGs in our analyses were also compared with these reports^5,10,11,65^.

### Validation of RNA sequencing results by qPCR

Seven differentially expressed genes were randomly selected (FC ≥ 2 or FC ≤ 0.5, and FDR < 0.05) between two Songliao and Landrace group for validation of RNASeq data using qPCR. Of these, four genes (PGM1, LDHA, PGK1, and ACACB) were significantly upregulated in Songliao group (FC ≥ 2), whereas three genes (ABCA4, LCN2, and VDR) were significantly downregulated in Songliao group (FC ≤ 0.5).

The relative expression abundances of DEGs (PGM1, LDHA, PGK1, ABCA4, LCN2, VDR, and ACACB) were validated using the Light Cycler® 480 Real-Time PCR System (Roche, USA). Total RNA was converted into cDNA using the Revert Aid™ First Strand cDNA Synthesis Kit (Thermo Fisher Scientific Inc, USA) following the manufacturer’s instructions. QPCR reactions were performed in a final volume of 20 μl with the Roche SYBR Green PCR Kit (Roche) according to the manufacturer’s protocol. Pig GAPDH was used as an internal standard to normalize the cDNA input. The primers used are described in Table S11. Triplicate qPCRs were performed for each cDNA and the average Ct was used for further analysis. Relative quantification values were calculated using the 2^−ΔΔCt^ method.

## Supplementary information

Supplementary Information

Dataset 1

Dataset 2

Dataset 4

Dataset 5

Dataset 6

Dataset 7

Dataset 8

## Data Availability

The deep sequencing data of total RNA were submitted to NCBI Sequence Read Archive (SRA) with accession number Bioproject: PRJNA234335 and Bioproject: PRJNA287471.
