# Supplementary material for: Comparative adipose transcriptome analysis digs out genes related to fat deposition in two pig breeds
Source: Sci Rep. 2019 Sep 9;9:12925. doi: 10.1038/s41598-019-49548-5 (PMC6733950; doi:10.1038/s41598-019-49548-5)
Supplement: Supplementary file 1 — Supplementary Information [file 41598_2019_49548_MOESM1_ESM.pdf]

Comparative adipose transcriptome analysis digs out genes related to fat deposition in two pig breeds

Kai Xing[1,2#], Kejun Wang[3#], Hong Ao[4], Shaokang Chen[5], Zhen Tan[2], Yuan Wang[2], Zhao Xitong[2], Ting Yang[2], Fengxia Zhang[2], Yibing liu[2], Hemin Ni[1], Xihui Sheng[1], Xiaolong Qi[1], Xiangguo Wang[1], Yong Guo[1*] and Chuduan Wang[2*]

[1]Animal Science and Technology College, Beijing University of Agriculture, Beijing 102206, China

[2]Key Laboratory of Animal Genetics, Breeding and Reproduction, Ministry of Agriculture, National Engineering Laboratory for Animal Breeding, College of Animal Science and Technology, China Agricultural University, Beijing 100193, China

[3]College of animal science and veterinary medicine, Henan Agricultural University, Zhengzhou, Henan, 450002， China

[4]State Key Laboratory for Animal Nutrition, Key Laboratory for Domestic Animal Genetic Resources and Breeding of the Ministry of Agriculture of China, Institute of Animal Science, Chinese Academy of Agricultural Sciences, Beijing 100193, China

[5]Beijing General Station of Animal Husbandry, Beijing 100125, China

[#] These authors contributed equally to this work.

[*] Corresponding authors

YG: y63guo@126.com

CW: cdwang@cau.edu.cn

Table S3. DEGs related to lipid metabolism and adipogenesis

Ensemble ID	Gene Symble	Gene title	Function	Log_2(Fold change)	FDR
ENSSSCG00000018080	ATP8	ATP synthase F0 subunit 8	Metabolic pathways、Oxidative phosphorylation and citric acid (TCA) cycle	-4.22	4.39E-13
ENSSSCG00000024476	CES3	carboxylesterase 3	Metabolism of lipids and lipoproteins、Biological oxidations、LDL-mediated lipid transport、Lipid digestion, mobilization, and transport	-2.64	1.80E-09
ENSSSCG00000006719	HSD3B1	hydroxy-delta-5-steroid dehydrogenase, 3 beta- and steroid delta-isomerase 1	Aldosterone synthesis and secretion、Glucocorticoid biosynthesis、Metabolism of lipids and lipoproteins、Metabolism of steroid hormones、Steroid hormone biosynthesis	-2.30	1.29E-08
ENSSSCG00000038521	CHAC1	ChaC glutathione specific gamma-glutamylcyclotransferase 1	Glutathione synthesis and recycling、Biological oxidation	-1.86	3.97E-06
ENSSSCG00000040464	LEP	leptin	Adipocytokine signaling pathway、AMPK signaling pathway、Incretin synthesis	-1.57	8.64E-04
ENSSSCG00000039862	TRIB3	tribbles pseudokinase 3	Metabolism of lipids and lipoproteins	-1.56	1.46E-04
ENSSSCG00000005638	LCN2	lipocalin 2	Members of this family transport small hydrophobic molecules such as lipids, steroid hormones and retinoids.	-1.46	1.91E-03
ENSSSCG00000036438	GPX3	glutathione peroxidase 3	Arachidonic acid metabolism、Glutathione metabolism	-1.45	2.65E-03
ENSSSCG00000000647	OLR1	oxidized low density lipoprotein receptor 1	PPAR signaling pathway	-1.40	4.93E-03
ENSSSCG00000030345	INPP5J	inositol polyphosphate-5-phosphatase J	Metabolism of lipids and lipoproteins	-1.38	2.78E-02
ENSSSCG00000003138	HSD17B14	hydroxysteroid 17-beta dehydrogenase 14	Metabolism of lipids and lipoproteins	-1.31	2.96E-03
ENSSSCG00000015355	DGKB	diacylglycerol kinase beta	Glycerolipid metabolism、Glycerophospholipid	-1.24	6.02E-03

			metabolism		
ENSSSCG00000006890	ABCA4	ATP binding cassette subfamily A member 4	active transport of a wide variety of substrates such as ions, sugars, lipids, sterols, peptides, proteins, and drugs	-1.23	2.72E-02
ENSSSCG00000010055	GGT5	gamma-glutamyltransferase 5	Metabolism of lipids and lipoproteins	-1.18	3.80E-02
ENSSSCG00000032843	AHRR	aryl-hydrocarbon receptor repressor	Fatty acid, triacylglycerol, and ketone body metabolism、Metabolism of lipids and lipoproteins、PPARA activates gene expression	-1.16	1.19E-02
ENSSSCG00000011297	ABHD5	abhydrolase domain containing 5	Lipid digestion, mobilization, and transport、Metabolism of lipids and lipoproteins、Regulation of lipolysis in adipocytes、triacylglycerol biosynthesis	-1.14	1.53E-02
ENSSSCG00000028204	TREH	trehalase	Metabolism of carbohydrates	-1.13	2.24E-02
ENSSSCG00000000660	A2M	alpha-2-macroglobulin	Lipid digestion, mobilization, and transport、Lipoprotein metabolism、Metabolism of lipids and lipoproteins	-1.13	3.43E-02
ENSSSCG00000003788	PTGER3	prostaglandin E receptor 3	Regulation of lipolysis in adipocytes	-1.09	2.40E-02
ENSSSCG00000011125	GATA3	GATA binding protein 3	Adipogenesis	-1.07	2.82E-02
ENSSSCG00000012889	CHKA	choline kinase alpha	Metabolism of lipids and lipoproteins	-1.06	2.76E-02
ENSSSCG00000002828	LPCAT2	lysophosphatidylcholine acyltransferase 2	Metabolism of lipids and lipoproteins	-1.05	3.35E-02
ENSSSCG00000016877	NNT	nicotinamide nucleotide transhydrogenase	Pyruvate metabolism and Citric Acid (TCA) cycle	1.00	4.65E-02
ENSSSCG00000006848	SLC25A24	solute carrier family 25 member 24	ATP transport、mitochondrial transport	1.01	4.87E-02
ENSSSCG00000016100	NDUFB3	NADH:ubiquinone oxidoreductase subunit B3	Oxidative phosphorylation、the citric acid (TCA) cycle and respiratory electron transport	1.03	4.61E-02
ENSSSCG00000009259	PAQR3	progestin and adipoQ receptor family member 3	Insulin receptor signalling cascade	1.03	4.99E-02
ENSSSCG00000037792	COX7A1	cytochrome c oxidase subunit 7A1	Oxidative phosphorylation	1.04	4.22E-02
ENSSSCG00000006107	PDP1	pyruvate dehyrogenase phosphatase	Pyruvate metabolism and Citric Acid (TCA) cycle	1.06	3.45E-02

		catalytic subunit 1			
ENSSSCG00000002374	DLST	dihydrolipoamide S-succinyltransferase	Citrate cycle (TCA cycle)	1.06	3.08E-02
ENSSSCG00000016516	ATP6V0A4	ATPase H+ transporting V0 subunit a4	Oxidative phosphorylation	1.06	4.09E-02
ENSSSCG00000004172	SLC2A12	solute carrier family 2 member 12	Transport of glucose and other sugars	1.07	3.54E-02
ENSSSCG00000023818	MRPS27	mitochondrial ribosomal protein S27	Mitochondrial translation	1.09	4.06E-02
ENSSSCG00000015435	NAMPT	nicotinamide phosphoribosyltransferase	Adipogenesis	1.09	3.65E-02
ENSSSCG00000035537	RUNX1	RUNX family transcription factor 1	Transport of glucose and other sugars	1.11	1.92E-02
ENSSSCG00000025901	LCLAT1	lysocardiolipin acyltransferase 1	Triacylglycerol biosynthesis	1.11	1.91E-02
ENSSSCG00000024015	FADS1	fatty acid desaturase 1	Biosynthesis of unsaturated fatty acids	1.14	1.96E-02
ENSSSCG00000022197	HS6ST1	heparan sulfate 6-O-sulfotransferase 1	Glycosaminoglycan biosynthesis	1.15	1.31E-02
ENSSSCG00000029515	PON3	paraoxonase 3	Metabolism of lipids and lipoproteins	1.16	1.92E-02
ENSSSCG00000033626	SREBF1	sterol regulatory element binding transcription factor 1	Adipogenesis、Insulin signaling pathway	1.16	3.61E-02
ENSSSCG00000005719	RAPGEF1	Rap guanine nucleotide exchange factor 1	Insulin signaling pathway	1.17	3.35E-02
ENSSSCG00000014149	MEF2C	myocyte enhancer factor 2C	Adipogenesis	1.20	3.28E-02
ENSSSCG00000027017	NR1D2	nuclear receptor subfamily 1 group D member 2	carbohydrate and lipid metabolism	1.21	4.87E-02
ENSSSCG00000014437	PPARGC1B	PPARG coactivator 1 beta	Metabolism of lipids and lipoproteins	1.22	8.79E-03
ENSSSCG00000008629	ROCK2	Rho associated coiled-coil containing protein kinase 2	Leptin signaling pathway	1.26	3.42E-02
ENSSSCG00000007507	PCK1	phosphoenolpyruvate carboxykinase 1	Glycolysis / Gluconeogenesis、Adipogenesis、PPAR signaling pathway、Pyruvate metabolism	1.27	5.32E-03
ENSSSCG00000013072	FADS2	fatty acid desaturase 2	Biosynthesis of unsaturated fatty acids、PPAR signaling pathway	1.29	1.03E-02

ENSSSCG00000015617	G0S2	G0/G1 switch 2	Metabolism of lipids and lipoproteins、Regulation of lipid metabolism	1.30	1.13E-02
ENSSSCG00000013399	TEAD1	TEA domain transcription factor 1	Metabolism of lipids and lipoproteins	1.30	3.40E-03
ENSSSCG00000006089	PTDSS1	phosphatidylserine synthase 1	Glycerophospholipid Biosynthetic Pathway	1.31	1.08E-02
ENSSSCG00000009889	ALDH2	aldehyde dehydrogenase 2 family member	Fatty acid degradation、Glycolysis / Gluconeogenesis、Pyruvate metabolism	1.34	4.94E-03
ENSSSCG00000001906	CYP1A1	cytochrome P450 family 1 subfamily A member 1	synthesis of cholesterol, steroids and other lipids	1.36	2.41E-02
ENSSSCG00000010301	PPP3CB	protein phosphatase 3 catalytic subunit beta	Glucagon signaling pathway	1.36	4.28E-03
ENSSSCG00000007873	ABCC6	ATP binding cassette subfamily C member 6	ABC transporters	1.36	6.72E-03
ENSSSCG00000030643	SELENOI	selenoprotein I	Glycerophospholipid biosynthesis 、Phosphatidylethanolamine (PE) biosynthesis	1.39	9.12E-04
ENSSSCG00000006328	RXRG	retinoid X receptor gamma	Adipocytokine signaling pathway 、PPAR signaling pathway	1.41	1.62E-02
ENSSSCG00000037559	ACOT11	acyl-CoA thioesterase 11	Fatty Acyl-CoA Biosynthesis 、 Triglyceride Biosynthesis	1.43	4.60E-04
ENSSSCG00000009937	ACACB	acetyl-CoA carboxylase beta	Fatty acid biosynthesis、	1.45	3.62E-03
ENSSSCG00000010947	FBP2	fructose-bisphosphatase 2	Glycolysis / Gluconeogenesis 、 Gluconeogenesis 、Glucose metabolism、Insulin signaling pathway	1.46	2.85E-03
ENSSSCG00000024814	PFKFB1	6-phosphofructo-2-kinase/fructose-2,6-biphosphatase 1	Gluconeogenesis、Glucose metabolism	1.48	5.58E-04
ENSSSCG00000015356	AGMO	alkylglycerol monooxygenase	Metabolism of lipids and lipoproteins	1.54	1.24E-03
ENSSSCG00000005229	VLDLR	very low density lipoprotein receptor	Lipid digestion	1.56	4.63E-03
ENSSSCG00000026554	PRXL2B	peroxiredoxin like 2B	Metabolism of lipids and lipoproteins	1.56	1.12E-04

ENSSSCG00000032498	IDH3A	isocitrate dehydrogenase (NAD(+)) 3 alpha	Citrate cycle (TCA cycle)、Pyruvate metabolism	1.59	9.69E-05
ENSSSCG00000038465	VAPA	VAMP associated protein A	Metabolism of lipids and lipoproteins	1.65	2.04E-04
ENSSSCG00000008803	ATP8A1	ATPase phospholipid transporting 8A1	Ion channel transport	1.67	1.38E-04
ENSSSCG00000018023	COX10	cytochrome c oxidase assembly factor heme A:farnesyltransferase COX10	Oxidative phosphorylation	1.69	1.78E-03
ENSSSCG00000032911	DUSP8	dual specificity phosphatase 8	Insulin receptor signalling cascade	1.75	8.99E-06
ENSSSCG00000003154	GYS1	glycogen synthase 1	Glucose metabolism、Glycogen synthesis、Insulin signaling pathway、Metabolism of carbohydrates	1.77	8.67E-06
ENSSSCG00000011363	PRKAR2A	protein kinase cAMP-dependent type II regulatory subunit alpha	Insulin signaling pathway、Glucagon signaling in metabolic regulation	1.77	5.25E-04
ENSSSCG00000004419	MFSD4B	major facilitator superfamily domain containing 4B	Transport of glucose and other sugars, bile salts and organic acids, metal ions and amine compounds	1.80	3.66E-05
ENSSSCG00000015949	SLC25A12	solute carrier family 25 member 12	Glucose metabolism、Gluconeogenesis	1.97	3.48E-07
ENSSSCG00000029811	PLCL2	phospholipase C like 2	lipid metabolic	2.01	8.85E-08
ENSSSCG00000014861	MOGAT2	monoacylglycerol O-acyltransferase 2	Triacylglyceride Synthesis	2.05	9.47E-08
ENSSSCG00000036614	PGP	phosphoglycolate phosphatase	Carbon metabolism、Glyoxylate and dicarboxylate metabolism	2.10	1.55E-08
ENSSSCG00000002873	GPI	glucose-6-phosphate isomerase	Gluconeogenesis、Glucose metabolism、Glycolysis	2.18	5.51E-09
ENSSSCG00000001852	IDH2	isocitrate dehydrogenase (NADP(+)) 2	Pyruvate metabolism and Citric Acid (TCA) cycle	2.18	8.51E-09
ENSSSCG00000015872	GPD2	glycerol-3-phosphate dehydrogenase 2	Metabolism of lipids and lipoproteins	2.23	5.97E-08
ENSSSCG00000038007	HACD1	3-hydroxyacyl-CoA dehydratase 1	Fatty acid biosynthesis	2.28	4.60E-04
ENSSSCG00000007744	PHKG1	phosphorylase kinase catalytic subunit gamma 1	Glucagon signaling pathway、Glucose metabolism、Insulin signaling pathway、Metabolism of carbohydrates	2.40	1.72E-05
ENSSSCG00000039425	BPGM	bisphosphoglycerate mutase	Glycolysis / Gluconeogenesis	2.56	2.60E-12

ENSSSCG00000012440	PGK1	phosphoglycerate kinase 1	Glycolysis / Gluconeogenesis、Glucose metabolism	2.57	5.24E-12
ENSSSCG00000014838	PGM2L1	phosphoglucomutase 2 like 1	Starch and sucrose metabolism	2.60	5.04E-10
ENSSSCG00000011698	GYG1	glycogenin 1	Glucose metabolism、glycogen biosynthesis、Starch and sucrose metabolism	2.66	3.29E-09
ENSSSCG00000002844	PHKB	phosphorylase kinase regulatory subunit beta	Glucose metabolism、Insulin signaling pathway、Metabolism of carbohydrates	2.69	3.96E-13
ENSSSCG00000010464	PPP1R3C	protein phosphatase 1 regulatory subunit 3C	Glucose metabolism、Insulin signaling pathway、Metabolism of carbohydrates	2.98	4.80E-16
ENSSSCG00000001930	PKM	pyruvate kinase M1/2	Carbon metabolism、Glucose metabolism、Glycolysis / Gluconeogenesis、Pyruvate metabolism	3.57	2.70E-07
ENSSSCG00000003812	PGM1	phosphoglucomutase 1	Glucose metabolism、Glycolysis / Gluconeogenesis、Metabolism of carbohydrates、Starch and sucrose metabolism	3.86	2.23E-21
ENSSSCG00000000694	GAPDH	glyceraldehyde-3-phosphate dehydrogenase	Carbon metabolism、Glycolysis / Gluconeogenesis	4.25	5.69E-08
ENSSSCG00000024018	SLC16A3	solute carrier family 16 member 3	Pyruvate metabolism and Citric Acid (TCA) cycle	4.27	1.44E-11
ENSSSCG00000013366	LDHA	lactate dehydrogenase A	Glycolysis / Gluconeogenesis、Pyruvate metabolism、The citric acid (TCA) cycle	4.41	4.00E-09
ENSSSCG00000016200	PRKAG3	protein kinase AMP-activated non-catalytic subunit gamma 3	key enzymes involved in regulating de novo biosynthesis of fatty acid and cholesterol	4.44	2.35E-21
ENSSSCG00000006872	AGL	amylo-alpha-1, 6-glucosidase, 4-alpha-glucanotransferase	Glucose metabolism、Metabolism of carbohydrates	4.47	2.50E-17
ENSSSCG00000012411	PHKA1	phosphorylase kinase alpha 1	Glucose metabolism、Insulin signaling pathway	4.72	1.83E-34
ENSSSCG00000032556	ALDOA	aldolase, fructose-bisphosphate A	Carbon metabolism、Glucose metabolism	5.08	5.16E-14
ENSSSCG00000021129	PFKM	phosphofructokinase, muscle	Galactose metabolism、Glucose metabolism、Glycolysis / Gluconeogenesis	6.13	9.86E-26

ENSSSCG00000016720	PGAM2	phosphoglycerate mutase 2	Carbon metabolism、Glucose metabolism、Glycolysis / Gluconeogenesis 、 superpathway of conversion of glucose to acetyl CoA and entry into the TCA cycle	8.32	2.91E-26
ENSSSCG00000017904	ENO3	enolase 3	Glucose metabolism、Carbon metabolism、Glycolysis / Gluconeogenesis	8.66	3.78E-28
ENSSSCG00000013022	PYGM	glycogen phosphorylase, muscle associated	Glucose metabolism 、Insulin resistance 、Starch and sucrose metabolism	9.15	1.41E-34

"+" and "–" in Log_2(Fold change) indicate the up- and down-regulated expression in Songliao group, respectively

Table S9. DEGs overlap QTLs.doc

Differentially expressed genes	Gene Symbl	QTL related to fat deposition and metobalism
ENSSSCG00000000810	AMIGO2	meat fatty acid content
ENSSSCG00000000811	-	meat fatty acid content
ENSSSCG00000001068	CAP2	leaf fat weight, subcutaneous adipose thickness, back fat thickness, back fat thickness,
ENSSSCG00000001493	PRIM2	meat fatty acid content,
ENSSSCG00000001500	-	back fat thickness, intramuscular fat content
ENSSSCG00000001683	POLH	backfat thickness
ENSSSCG00000001852	IDH2	shoulder subcutaneous fat thickness
ENSSSCG00000001873	CSPG4	meat fatty acid content
ENSSSCG00000002285	GPHN	back fat thickness
ENSSSCG00000003582	SMPDL3B	backfat thickness
ENSSSCG00000003693	MYOM1	backfat thickness
ENSSSCG00000003768	-	back fat thickness
ENSSSCG00000004022	RPS6KA2	intramuscular fat content
ENSSSCG00000004367	POPDC3	dressed carcass fat content, back fat thickness
ENSSSCG00000005229	VLDLR	abdominal fat weight, back fat weight
ENSSSCG00000005713	-	dressed carcass fat percentage
ENSSSCG00000005905	SLC39A4	backfat thickness
ENSSSCG00000006089	-	meat fatty acid content
ENSSSCG00000006137	WWP1	meat fatty acid content
ENSSSCG00000006160	STMN2	meat unsaturated fatty acid content, backfat at last rib
ENSSSCG00000006218	PDE7A	backfat at first rib
ENSSSCG00000006338	DDR2	meat fatty acid content
ENSSSCG00000006542	-	meat fatty acid content, meat unsaturated fatty acid content,
ENSSSCG00000006872	-	back fat thickness, meat fatty acid content
ENSSSCG00000008446	SIX2	average backfat thickness
ENSSSCG00000008991	FRAS1	back fat thickness
ENSSSCG00000009125	-	meat fatty acid content
ENSSSCG00000009192	-	intramuscular fat content, meat fatty acid content
ENSSSCG00000009693	XKR6	average backfat thickness
ENSSSCG00000010603	NEURL1	meat fatty acid content
ENSSSCG00000011363	PRKAR2A	backfat thickness
ENSSSCG00000011367	ARIH2	average backfat thickness
ENSSSCG00000012371	AR	back fat thickness
ENSSSCG00000013022	PYGM	average backfat thickness
ENSSSCG00000013043	MACROD1	meat fatty acid content, subcutaneous adipose thickness, intramuscular fat content, back fat thickness

ENSSSCG00000013101	MS4A4A	average backfat thickness
ENSSSCG00000015402	CACNA2D1	belly fat area
ENSSSCG00000015617	G0S2	meat polyunsaturated fatty acid content
ENSSSCG00000015917	XIRP2	intramuscular fat content
ENSSSCG00000016685	-	meat fatty acid content
ENSSSCG00000016722	UBE2D4	meat lipid content
ENSSSCG00000016956	-	meat fatty acid content
ENSSSCG00000017282	-	meat fatty acid content, fat percentage in carcass
ENSSSCG00000018007	LOC396711	intramuscular fat content,
ENSSSCG00000020785	DES	intramuscular fat content
ENSSSCG00000022945	-	average backfat thickness
ENSSSCG00000023296	-	meat fatty acid content
ENSSSCG00000026164	SOHLH2	average backfat thickness
ENSSSCG00000027992	MST1R	meat oleic acid content, meat monounsaturated fatty acid content
ENSSSCG00000028052	OBSL1	intramuscular fat content
ENSSSCG00000028172	FAM78A	dressed carcass fat percentage
ENSSSCG00000028420	EIF4E	meat fatty acid content
ENSSSCG00000029441	-	intramuscular fat content, total body fat weight
ENSSSCG00000030643	SELENOI	average backfat thickness

Table S10. The result compared with previous similar reports

Previous similar reports	Common DEGs	Log_2FC in this study	Log_2FC in previous reports	Description
Jeju Native Pig (obesity) VS. Berkshire (lean)	COL11A2	-1.02	-93.49	collagen type XI alpha 2 chain
	PPL	-1.56	5.92	periplakin
	CCL4	1.40	-11.20	CCR-like protein 4
	FMO1	1.56	-4.03	flavin containing monooxygenase 1
	ITGA8	1.57	-4.86	integrin alpha 8
	LCN2	1.78	-6.95	lipocalin 2
Korean native pig (obesity) VS. Yorkshire (lean)	CPEB4	-2.63	1.87	cytoplasmic polyadenylation element binding protein 4
	DBNDD2	-1.05	1.49	dysbindin domain containing 2
	FBP2	-1.24	1.58	Fat body protein 2
	NR4A3	-2.04	4.70	nuclear receptor subfamily 4 group A member 3
	PDE4D	-2.51	-1.56	phosphodiesterase 4D
	SLC25A4	-1.63	-2.03	solute carrier family 25 member 4
	TPM2	-3.80	2.17	tropomyosin 2
	A2M	1.18	4.11	alpha-2-macroglobulin
	C4BPA	1.07	-2.43	complement component 4 binding protein alpha
	CES3	2.70	1.46	carboxylesterase 3
	GMFG	1.01	1.62	glia maturation factor gamma
Rongchang piglet (obesity) VS. Landrace (lean)	FOS	-2.18	6.47	FBJ osteosarcoma oncogene
	CLDN7	-1.41	2.78	claudin 7
	RCAN1	-1.88	3.77	regulator of calcineurin 1
	TRIM63	-3.80	2.68	tripartite motif containing 63
	CLEC7A	1.21	8.99	C-type lectin domain family 7, member a
	HES6	-1.06	2.82	hes family bHLH transcription factor 6
	OLR1	1.30	3.14	oxidized low density lipoprotein receptor 1
	S100A4	1.51	4.44	S100 calcium binding

				protein A4
Italian Large White pigs with high backfat thickness (obesity) VS. Italian Large White pigs with low backfat thickness (lean)	HSPB8	-5.10	-.181	heat shock protein family B (small) member 8
	GMFG	1.01	1.23	glia maturation factor gamma
	LYZ	2.41	2.03	lysozyme
	OLR1	1.30	1.26	oxidized low density lipoprotein receptor 1
	PADI2	1.02	1.6	peptidyl arginine deiminase 2
	ST14	1.16	1.79	suppression of tumorigenicity 14
	STMN2	1.32	1.85	stathmin-like 2

Table S11. Forward and reverse primers used for qPCR validation of 7 genes from RNASeq.

Gene		Primer Sequences for qPCR (5’-3’)	Amplicon length (bp)
PGM1	F	GATCCTGTGGACGGAAGCAT	122
	R	ATGTACAGTCGGATGGTGGC	
LDHA	F	AGTGCACTCCCGATTCCTTT	129
	R	AACAGCACCAACCCCAACAA	
PGK1	F	GCTGGACGTGAAGGGAAAGA	140
	R	CTGACTTGGCTCCGTTGTCT	
ABCA4	F	AAATGACGCACGACTTCCCT	142
	R	AACCTGGAGGCACCTATCCT	
LNC2	F	GTCAGGTGGACGGACCATAAA	126
	R	GCCACAGGAGACTTAGGGC	
VDR	F	TGGTTGGAAGTGTCTGGGAG	140
	R	GGCACATTCCGGTCAAAGTC	
ACACB	F	CCCGACCATGTTTGTCCTCA	145
	R	GGTGAGGCGGTAACTGTTGA	
GAPDH	F	GGGCATGAACCATGAGAAGT	230
	R	AAGCAGGGATGATGTTATGG	
